# *Pneumocystis jirovecii* in General Population

**DOI:** 10.3201/eid1102.040487

**Published:** 2005-02

**Authors:** Francisco J. Medrano, Marco Montes-Cano, Manuel Conde, Carmen de la Horra, Nieves Respaldiza, Antonia Gasch, Maria J. Perez-Lozano, Jose M. Varela, Enrique J. Calderon

**Affiliations:** * Virgen del Rocío University Hospital, Seville, Spain

**Keywords:** Pneumocystis, Pneumocystis jirovecii, epidemiology, polymerase chain reaction, community survey, research

## Abstract

The possible presence of *Pneumocystis* among healthy adults was examined by detecting *Pneumocystis jirovecii*–specific DNA in prospectively obtained oropharyngeal wash samples from 50 persons without underlying lung disease or immunosuppression. *Pneumocystis* carriage, defined by detecting *Pneumocystis* DNA by nested polymerase chain reaction in 2 independent analyses plus successful mitochondrial large subunit ribosomal RNA typing by direct sequencing, was found in 20% of cases. All carriers were asymptomatic, anti-HIV negative, and had normal total lymphocyte and CD4+ cell counts. A second sample obtained in the 6-month follow-up was positive in 2 of 9 available carriers. Genotype analysis showed different polymorphisms; 85A/248C (40%) and 85C/248C (30%) were most frequently observed. This study provides the first evidence that *P. jirovecii* DNA can be frequently detected in the respiratory tract of immunocompetent adults, which agrees with the hypothesis that the general population could be a reservoir and source of this infection.

## Abstract 2

*P. jirovecii* colonization can be frequently detected in immunocompetent adults, which suggests that the general population could be a source of this infection.

*Pneumocystis jirovecii* (formerly known as *Pneumocystis carinii* f. sp. *hominis*) (*1*) is the causative agent of *Pneumocystis* pneumonia (PCP), one of the most frequent and severe opportunistic infections in immunocompromised patients (*2*). *Pneumocystis* organisms represent a large group of species of atypical fungi with universal distribution and pulmonary tropism, and each species has a strong specificity for a given mammalian host species (*3**,**4*).

Despite the advances made in understanding human *Pneumocystis* infection, many aspects about its epidemiology and natural history remain unclear. Serologic studies have shown that specific antibodies to the pathogen can be detected in most children early in life (*5**–**7*), which indicates frequent exposure to this organism. Based on this finding, disease in immunocompromised persons has long been thought to result from reactivation of latent infection acquired in childhood. However, animal and human studies have shown that elimination of *Pneumocystis* often occurs after infection (*8**–**9*), which implies that the persistence of latent organisms is limited.

Alternatively, the possibility that *Pneumocystis* can be transmitted from person to person has been raised after the reports of cluster outbreaks of PCP among solid-organ transplant and oncology patients (*10**,**11*). Evidence supporting the active or de novo airborne acquisition of the organism from human sources has accumulated in the last few years, including evidence for different *Pneumocystis* genotypes in different episodes of PCP in the same patient (*12**,**13*). Also, *Pneumocystis* DNA was detected in the upper respiratory tract of healthy participants after close contact with patients with PCP (*14**–**16*) and in air samples from the rooms of PCP patients (*17**,**18*). *Pneumocystis* has also been found in immunosuppressed patients without PCP (*19*); these situations have been described as *Pneumocystis* colonization or carriage.

PCP patients, immunodeficient carriers, or transiently parasitized immunocompetent persons have been hypothesized to play a role as sources of *Pneumocystis* infection (*4*). Although some earlier studies failed to detect the organism in postmortem lung samples or bronchoalveolar samples from immunocompetent adults (*20**,**21*), a recent report indicates that *Pneumocystis* DNA can be frequently detected in healthy infants (*22*).

The ability to detect *Pneumocystis* in normal, healthy persons is due to the development of more sensitive methods. *Pneumocystis* can now be detected in respiratory samples obtained by noninvasive methods using immunofluorescence staining and polymerase chain reaction (PCR) (*23**,**24*). By using these methods, *Pneumocystis* carriage was found in 10% to 40% of immunocompetent patients with different chronic lung diseases (*25**,**26*). From an epidemiologic point of view, this high prevalence is difficult to determine if PCP or colonized immunosuppressed patients and their close contacts are the only sources of infection, since the persistence of latent organism in lung appears to be time-limited (*8**,**9*). We tested the hypothesis that in a normal community environment healthy adults can be transiently colonized by *Pneumocystis*, and these persons play a role in the persistence of the organism in the human ecosystem. Identifying *Pneumocystis* sources is essential to developing proper measures to prevent a disease that still causes substantial illness and death among immunosuppressed patients. This study attempts to determinate whether *P. jirovecii* can be detected in the general normal, healthy population.

## Methods

### Study Population

This prospective study included persons who 1) had not been exposed to patients in a hospital environment within the year before the study or 2) had not been diagnosed with or were not suspected to have chronic lung disease, neoplasm, or immunosuppression of any cause. The first 50 persons evaluated in the Occupational Health Service of the Virgen del Rocío University Hospital from February to July 2003 who had not been excluded by the above criteria were enrolled in this study.

The mean age of persons in this study group was 33.9 ± 9.45 years. Nineteen (31.6%) were male. Distribution according to professional standing was 28 (56%) newly employed physician residents, 13 (26%) university or common services staff members, and 9 (18%) administrative staff.

Each participant underwent a clinical-epidemiologic examination, and oropharyngeal samples were collected for analysis in the Occupational Health Service, a building located outside the hospital environment. Demographic variables, underlying medical conditions, habits, and antimicrobial therapy were recorded by using a standardized form. Informed consent was obtained from participants. The study protocol was designed and performed according to the Helsinki Declaration and was approved by the ethics committee, Virgen del Rocío University Hospital, Seville, Spain.

For all *Pneumocystis* carriers, a complete clinical and biologic evaluation was performed, including physical examination, chest x-ray, conventional blood work, anti-HIV serologic examination, and peripheral blood lymphocyte subsets analyses. Volunteers who were designated *P. jirovecii* carriers were reexamined after 6 months, when oropharyngeal samples were again obtained.

### Case Definition

A *Pneumocystis* carrier was defined as a person who met all of the following conditions: 1) no clinical history of PCP, 2) respiratory specimen with detectable *P. jirovecii* DNA by nested PCR in 2 independent analyses, and 3) successful mitochondrial large subunit ribosomal RNA (mtLSU-rRNA) typing of the respiratory specimen by direct sequencing at least once. Persons who did not meet these criteria were considered *Pneumocystis*-negative.

### Sampling and Detecting *P. jirovecii*

Oropharyngeal wash samples were obtained by gargling with 10 mL of sterile physiologic serum (0.9% NaCl) for a period of 1 min. Samples were centrifuged at 2,900 x *g* for 5 min and kept frozen at –20°C until DNA was extracted.

After digestion with proteinase K at 56°C for 2 h, DNA from 2 aliquots of each oropharyngeal wash sample was extracted on 2 days by using a commercial kit from Qiagen (Hilden, Germany). Sham extractions, carried out in parallel with the processing of samples, were also included to control for contamination in the DNA extraction step. The purified DNA was used as a template to amplify the region containing mtLSU-rRNA by nested PCR, as described elsewhere (*27**,**28*). The sensitivity of this nested PCR assay is 1 organism/mL. Briefly, in the first amplification round, the external primers pAZ102-E (5´-GAT GGC TGT TTC CAA GCC CA-3´) and pAZ102-H (5´-GTG TAC GTT GCA AAG TAC TC-3´) were used. This amplification yields a 346–base pair (bp) fragment. The second round of amplification used the primers pAZ102-X (5´-GTG AAA TAC AAA TCG GAC TAG G-3´) and pAZ102-Y (5´-TCA CTT AAT ATT AAT TGG GGA GC-3´) and yielded a 260-bp product. Forty cycles of amplification were carried out for both rounds.

The amplification products were analyzed by electrophoresis on a 1.5% agarose gel containing ethidium bromide, and the bands were visualized by UV light. To prevent false-positives from contamination, pipettes with filters were used in all manipulations. DNA extraction, preparation of the reaction mixture, PCR amplification, and detection were performed in different areas under a laminar flow hood. To detect any cross-contamination, all PCRs were performed with negative controls and sterile water.

The products from nested PCR amplification were purified by using Sephacryl S-400 columns (Amersham Pharmacia Biotech AB, Uppsala, Sweden) and reamplified by using ABI Prism dRhodamine Terminator Cycle Sequencing Ready Reaction Kit (PE Applied Biosystems, Foster City, CA, USA). Then, for each reaction, 5 µL of PCR product, 4 µL terminator-ready reaction mix, and 3 pmol of primer were added. The extension products were purified by ethanol precipitation to remove excess dye terminators. Each sample pellet was resuspended in 12.5 µL of template suppression reagent and heated at 95°C for 3 min for denaturation. Electrophoresis was carried out on the ABI prism 310 sequencer (PE Applied Biosystems) according to manufacturer's recommendations. The sequenced DNA fragments were analyzed by using Sequence Navigator v.1.0.1 (PE Applied Biosystems).

In the specimens of carriers, the *P. jirovecii* dihydropteroate synthase (DHPS) locus was analyzed by PCR restriction fragment length polymorphism (RFLP), as previously described (*28*). In brief, the single-copy gene of DHPS was amplified by the primers DHPS-3 (5´-GCG CCT ACA CAT ATT ATG GCC ATT TTA AAT C-3´) and DHPS-4 (5´-GGA ACT TTC AAC TTG GCA ACC AC-3´) by using a touchdown-PCR protocol, yielding a 370-bp fragment. The PCR product was divided into 3 aliquots. One was used to confirm the presence of a 370-bp fragment from the DHPS gene. The second and third aliquots were used to identify wild-type sequences versus mutations in codons 55 and 57 by RFLP with AccI and HaeIII (Roche Diagnostics, Mannheim, Germany), respectively. When the mutation is present, a 370-bp band appears. After RFLP in wild-type samples, bands appear at 229 bp and 141 bp with AccI and at 221 bp and 149 bp with HaeIII.

### Laboratory Studies

Peripheral blood lymphocyte subsets were determined by using a flow cytometer (Cytoron Absolute, Ortho, Raritan, NJ, USA) after incubation with monoclonal antibodies OKT3, OKT4, and OKT8 (Ortho). Serum anti-HIV antibodies were determined by a commercial enzyme-linked immunosorbent assay (ELISA) (Multispot HIV-1/HIV-2 rapid test, BioRad, Hercules, CA, USA). The results were interpreted according to the manufacturer's recommendations.

### Statistical Analysis

The chi-square test was used for assessing differences between proportions. Results were considered significant at p < 0.05. Statistical analyses were performed by using the Statistical Package for Serial Studies for personal computers (SPSS version 12, SPSS Inc., Chicago, IL, USA).

## Results

*P. jirovecii* DNA in 12 of 50 samples was successfully amplified twice by nested PCR. The mtLSU-rRNA fragment locus was successfully typed in 10 of the 12 samples in which mtLSU-rRNA had been amplified. Thus, *P. jirovecii* carriage was detected in 20% of the participants. To assess the reproducibility and consistency of results, serial samples were obtained with a 2-day interval in 5 participants (1 carrier and 4 noncarriers). In all of them, consistent positive and negative patterns of results were obtained (positive PCR test after a positive result and negative PCR test after a negative result).

The DHPS primer sets amplified a 370-bp band in 7 (70%) of 10 carriers. In all positive specimens with the DHPS-based PCR assay, the RFLP technique identified a wild DHPS genotype (Table).

**Table:** Epidemiologic, biologic, and microbiologic features of healthy *Pneumocystis* carriers*

Participant (age [y], sex)†	Profession	Alcohol intake >40 g/day	Smoking habit	Total lymphocyte count (cells/mL)	CD4+ cell count (cells/mL)	mtLSU-rRNA genotype (nucleotide position: identity)	DHPS genotype (codon position: identity) by PCR-RFLP assay	PCR result at month 6 of follow-up
1 (28, F)	Administrative	No	No	2,327	653	3 (85:T/248:C)	1 (55:Thr/57:Pro)	+
2 (42, F)	Administrative	No	No	2,455	1,168	1 (85:C/248:C)	1 (55:Thr/57:Pro)	–
3 (43, F)	Administrative	No	No	NA	NA	4 (85:C/248:T)	Not amplified	–
4 (40, M)	Administrative	Yes	No	1,528	520	3 (85:T/248:C)	Not amplified	–
5 (41, F)	New resident	No	No	NA	NA	2 (85:A/248:C)	1 (55:Thr/57:Pro)	NA
6 (27, F)	New resident	No	No	1,354	550	2 (85:A/248:C)	1 (55:Thr/57:Pro)	–
7 (32, F)	New resident	No	No	2,300	604	1 (85:C/248:C)	1 (55:Thr/57:Pro)	–
8 (40, M)	University staff	Yes	Yes	1,730	924	2 (85:A/248:C)	1 (55:Thr/57:Pro)	+
9 (26, M)	University staff	Yes	Yes	3,602	924	2 (85:A/248:C)	1 (55:Thr/57:Pro)	–
10 (42, M)	University staff	No	No	1,518	432	1 (85:C/248:C)	Not amplified	–

*mtLSU-rRNA, mitochondrial large subunit ribosomal RNA; DHPS, dihydropteroate synthase; PCR, polymerase chain reaction; RFLP, restriction fragment length polymorphism; F, female; M, male; +, positive result; –, negative result; NA, not available. †Chest radiograph results were normal for all participants, except participant 5, for whom results were not available, and participant 9, who showed an apical cystic bullae.

Results of physical examination were normal for *Pneumocystis* carriers. All were anti-HIV negative and had normal total lymphocyte and CD4+ cell counts. Chest radiographs were normal in 8 participants, and 1 participant had an apical cystic bullae (Table). Only 1 person had taken steroids for a brief period in the 6 months before the study. No differences were detected due to age, sex, profession, alcohol intake, and smoking habit between *P. jirovecii* carriers and noncarriers.

Five known mtLSU-rRNA types are described for this *Pneumocystis* gene locus (*15*); 4 genotypes were isolated in the current study. Genotypes at this locus were identified on the basis of polymorphisms at nucleotide positions 85 and 248. Genotype 2 (85:A/248:C) was observed in 4 cases, genotype 1 (85:C/248:C) in 3 cases, genotype 3 (85:T/248:C) in 2 cases, and genotype 4 (85:C/248:T) in the remaining case (Table).

During the follow-up, all carriers were asymptomatic for pulmonary disease. *Pneumocystis* DNA was detected by PCR in a second oropharyngeal wash obtained 6 months after the first in 2 (22.2%) of the 9 available carriers. Neither sample was typed because of insufficient quantity of PCR product.

## Discussion

This study on immunocompetent healthy adults documents that *P. jirovecii* DNA can be detected by sensitive DNA amplification techniques by using noninvasive sampling of the respiratory tract. DNA detection does not establish the existence of infectious intact organisms. However, in animal models, detecting *Pneumocystis* DNA in nasal and oral samples is a good indicator that the organism is in the lung (*29*). Also, experiments show that *Pneumocystis* organisms can replicate in the lung alveolus of immunocompetent hosts and remain infectious (*30*). Thus, our results agree with the hypothesis that the general human population could play an important role as a reservoir and source of *P. jirovecii* infection and support the saprophytic nature of this pathogen in humans.

An important finding of this study is that *Pneumocystis* DNA was not detected in >75% of the immunocompetent colonized adults within 6 months, which suggests the possible transience of the carrier state in healthy persons. This observation agrees with previous reports that show that most immunocompetent healthcare workers who were colonized with the pathogen cleared the infection (*15**,**16*).

The number of persons examined in this study sufficiently demonstrated that *P. jirovecii* is an organism frequently found in healthy adults in the normal community. Since participants were all affiliated in some way with the Virgen del Rocío University Hospital in Seville, Spain, a broader group of healthy adults would need to be examined to estimate the prevalence of the carriers in the general population. Carrying out the study in a hospital could have somewhat biased the results, although we excluded persons with prior exposure to patients within the hospital and collected samples in a building outside the hospital.

The accepted current diagnostic standard for *Pneumocystis* infection is the direct demonstration of the stained microorganism in respiratory samples. Techniques based on PCR amplification of specific genome regions that provide high sensitivity are now widely used to diagnose several infectious diseases. These technical advances have allowed us to detect infections with samples obtained by noninvasive methods and in samples with low pathogen load. Different studies involving both sputum and bronchoalveolar lavage specimens have demonstrated the higher sensitivity of these techniques compared to conventional staining and monoclonal antibody immunohistochemical techniques (*31**,**32*). The main drawback to PCR is the possibility of false-positives (usually because of contamination) and the absence of rapid culture methods to confirm the PCR amplification results obtained. We avoided potential false-positives by adopting stringent precautionary measures and by examining the PCR signal from 2 different genes of *P. jirovecii*.

The mtLSU-rRNA gene was selected for genotyping because it has a high degree of genetic conservation and is useful for detecting intraspecific differences between populations (*33*). The allelic frequency distribution patterns at this gene seen in the present study are similar to those reported in AIDS-associated PCP cases in a large study conducted in 5 cities in the United States (*33*). In that study, *P. jirovecii* genotypes were correlated with the place of diagnosis, rather than the person's place of birth. Our results support the concept of a community source of the infectious agents. Furthermore, an epidemiologic study was recently performed in patients in Spain with various pulmonary diseases. By also analyzing mtLSU-rRNA types, we found a high prevalence of genotype 1 (45%) and genotype 3 (40%) and lower of genotype 2 (10%) (*28*). In contrast, in the present study, genotype 2 was the most prevalent, whereas it was low in the pulmonary patient study, and genotype 3 was more frequently found in pulmonary patients than in normal healthy adults. Patients with pulmonary disorders may have a greater susceptibility to genotype 3.

In our study, the rate of carriers identified as having the DHPS gene is 70%. The low amplification rate obtained is perhaps related to a low pathogen load in the samples. However, this rate is similar to that reported for AIDS patients with PCP in previously published studies (*33*).

In the last decade, PCR technologies have shown that immunocompromised patients without PCP can be subclinically infected with *Pneumocystis* (*19*). In addition, a high prevalence of *Pneumocystis* is seen among immunocompetent patients with chronic pulmonary disorders (*25**,**26**,**34*), patients with small-cell lung carcinoma (*35*), or pregnant women (*36*). The pathogen has been detected in immunocompetent contacts of patients with PCP and in immunocompetent healthcare workers, whether or not they had contact with immunocompromised patients (*15**,**16*). Also, immunocompetent animal model hosts are commonly transiently colonized; *Pneumocystis* can replicate actively in their lungs and can be transmitted to another host (*30**,**37*). Moreover, postmortem lung screening using conventional staining for *Pneumocystis* showed small numbers of the organism in the lungs of immunocompetent individuals (*38*).

Thus, substantial evidence exists of *Pneumocystis* colonization of healthy persons, and our findings are consistent with these observations. Most previous studies that failed to find the organism on autopsy (*21*) or in respiratory samples (*14**,**29**,**31**,**36**,**39*) from immunocompetent persons were performed on only a few persons. In some cases, failure was probably related to the use of either single PCR (*31*) or a less sensitive PCR (*39*). In others, failure could be related to the use of sputum or nasal secretions (*14**,**29**,**36*) that may have had lower numbers of organisms than in the oropharyngeal wash we analyzed or because of different procedures used for analyses (*29*). Protocols for acquiring and processing respiratory samples and analytical probes and methods should be standardized to enable better comparisons between studies performed in different laboratories.

In summary, immunocompetent healthy adults might harbor short-lived infections that could be transmitted to other immunocompetent host in whom a transient infection can develop. Similarly, infants can become infected and a primary infection can develop, and immunosuppressed, susceptible people can become infected and clinical PCP can develop. Today, we know that human pneumocystosis is anthroponotic. Our findings may suggest that healthy adults represent a new dynamic reservoir and source of infection for human *Pneumocystis* species. Immunocompetent carriers in community ecosystems might present a public health issue that merits further research.
